# Comparison of coated and uncoated trace elements on growth performance, apparent digestibility, intestinal development and microbial diversity in growing sheep

**DOI:** 10.3389/fmicb.2022.1080182

**Published:** 2022-12-20

**Authors:** Jia Zhou, Yifan Ren, Xiao Wen, Shuangming Yue, Zhisheng Wang, Lizhi Wang, Quanhui Peng, Rui Hu, Huawei Zou, Yahui Jiang, Qionghua Hong, Bai Xue

**Affiliations:** ^1^Key Laboratory of Low Carbon Culture and Safety Production in Cattle in Sichuan, Animal Nutrition Institute, Sichuan Agricultural University, Chengdu, China; ^2^Department of Bioengineering, Sichuan Water Conservancy Vocational College, Chengdu, China; ^3^College of Animal Science and Technology, Sichuan Agricultural University, Chengdu, China; ^4^Yunnan Animal Science and Veterinary Institute, Kunming, China

**Keywords:** trace elements, sheep, growth performance, intestinal development, gut microbiota

## Abstract

The suitable supplement pattern affects the digestion and absorption of trace minerals by ruminants. This study aimed to compare the effects of coated and uncoated trace elements on growth performance, apparent digestibility, intestinal development and microbial diversity in growing sheep. Thirty 4-month-old male Yunnan semi-fine wool sheep were randomly assigned to three treatments (*n* = 10) and fed with following diets: basal diet without adding exogenous trace elements (CON), basal diet plus 400 mg/kg coated trace elements (CTE, the rumen passage rate was 65.87%) and basal diet plus an equal amount of trace elements in uncoated form (UTE). Compared with the CON group, the average daily weight gain and apparent digestibility of crude protein were higher (*P* < 0.05) in the CTE and UTE groups, while there was no difference between the CTE and UTE groups. The serum levels of selenium, iodine and cobalt were higher (*P* < 0.05) in the CTE and UTE groups than those in the CON group, the serum levels of selenium and cobalt were higher (*P* < 0.05) in the CTE group than those in the UTE group. Compared with the CON and UTE groups, the villus height and the ratio of villus height to crypt depth in duodenum and ileum were higher (*P* < 0.05) in the CTE groups. The addition of trace minerals in diet upregulated most of the relative gene expression of *Ocludin*, *Claudin-1*, *Claudin-2*, *ZO-1*, and *ZO-2* in the duodenum and jejunum and metal ion transporters (*FPN1* and *ZNT4*) in small intestine. The relative abundance of the genera *Christensenellaceae R-7 group*, *Ruminococcus 1*, *Lachnospiraceae NK3A20 group*, and *Ruminococcaceae* in ileum, and *Ruminococcaceae UCG-014* and *Lactobacillus* in colon was higher in the CTE group that in the CON group. These results indicated that dietary trace mineral addition improved the growth performance and intestinal development, and altered the structure of intestinal bacteria in growing sheep. Compared to uncoated form, offering trace mineral elements to sheep in coated form had a higher absorption efficiency, however, had little effect on improving growth performance of growing sheep.

## 1 Introduction

Trace elements such as iron (Fe), copper (Cu), manganese (Mn), zinc (Zn), selenium (Se), iodine (I), and cobalt (Co) are an essential class of substances for animals provided primarily by food sources. Although the mass proportion in animal body is less than 0.01%, trace elements play an indispensable role in maintaining their normal physiological activities, such as hemopoiesis, immune response, energy metabolism, enzyme activity and reproductive function (Rabiee et al., 2010; Overton and Yasui, 2014). A deficiency of any of these trace minerals could lead to a negative impact on the health and productivity of livestock (Spears and Weiss, 2008). Commonly, inorganic salts of trace elements, such as sulfate, oxide, carbonate and chloride are supplemented in diets to meet the requirement of livestock for trace elements. However, for ruminants, the ionic bonds in inorganic salts usually dissociate as they flow through the digestive tract, allowing undesirable interactions with other molecules and preventing absorption, thus reducing the bioavailability and efficiency of trace elements (Spears, 1996, 2003; Samarin et al., 2022). For example, sulfates are first reduced to sulfides by rumen microbes, which react with molybdate to form thiomolybdate and then react with cupric ion to generate un-digestible compounds, thereby limiting the absorption of dietary Cu (Dick et al., 1975). The efficiency of absorption of Cu and Se by ruminants is much lower than that of non-ruminants (Spears, 2003). Furthermore, in some scenarios, rumen fermentation was affected by feeding conventional mineral salts, as was ruminal digestibility of nutrients (Genther and Hansen, 2015; Faulkner and Weiss, 2017; Arce-Cordero et al., 2020). To improve the availability of trace elements, the addition of coated trace elements (slow release in the rumen) to the diet was a practicable strategy (Abdelrahman et al., 2017; Arce-Cordero et al., 2020; Potier et al., 2022).

There were a series of reports on the effective application of coated trace elements in ruminants. Previous studies reported that supplementation of coated slow-release trace elements at late gestation increased the content of Zn and Se in plasma and milk of ewes, and enhanced the birth and weaning weight of lambs (Abdelrahman et al., 2017; Aliarabi et al., 2019). Supplementation of coated sustained-release trace minerals also improved the milk performance and altered rumen microbial structure of lactating yaks (Zhao et al., 2022). Compared with the addition of copper sulfate in diets, coated copper sulfate addition improved lactation performance and apparent nutrient digestibility in dairy cows (Wang C. et al., 2021), increased the average daily gain and stimulated rumen enzyme activity in Holstein bulls (Wu et al., 2021). The addition of an equal amount of coated sodium selenite contributed to improve the milk production of dairy cows (Zhang et al., 2020), improved the ruminal fermentation and nutrient digestibility of dairy bulls (Liu et al., 2020), compared to uncoated sodium selenite. However, to our knowledge, there is very little information comparing uncoated and coated compound trace elements in ruminants.

The improved bioavailability of trace elements is beneficial to exert their biological functions. More than 50% of known enzymes have at least one metal as a cofactor, of which zinc is the most common, followed by iron and manganese (Andreini et al., 2008). Most trace minerals are also involved in redox reactions, as free radicals are able to interact with metal-binding superoxide dismutase and break down into less or less toxic substances, indicating that trace minerals play an important role in alleviating oxidative stress (Pajarillo et al., 2021). Dietary zinc oxide supplementation enhanced intestinal morphological and functional development by increasing antioxidant capacity and maintaining mucosal barrier integrity in weaned piglets (Zhu et al., 2017, 2017). Besides, trace elements such as Fe, Mn, Zn, Se and Cu have been revealed to regulate the gut microbiota in different animals or *in vitro* (Dostal et al., 2015; Zhang et al., 2019; Foong et al., 2020; Kociova et al., 2020; Wang et al., 2020). The regulation of the gut barrier and immune response by selenium intake has been attributed to modulation of the gut microbiota (Kasaikina et al., 2011). We suspected that the coated trace elements had better biological activity on growth performance and intestinal development in sheep compared to uncoated trace elements. Thus, the ultimate objective of this study was to compare the effects of coated and uncoated trace elements on growth performance, apparent digestibility, intestinal development, and microbial diversity in ileum and colon of growing sheep.

## 2 Materials and methods

### 2.1 Approval by the ethics committee

All animals in this study were treated according to the Chinese Guidelines for Animal Welfare and the protocol was approved by the Experimental Animal Committee of Animal Nutrition Institute, Sichuan Agricultural University (Approval number: #SCAUAC2019-36).

### 2.2 Experimental design, diets, and animals

**Exp. 1.** The experiment was performed at the farm of Kunming Yixingheng Livestock Technology Co., Ltd. (Yunnan, China; 25.07°N, 102.55°E, and 2,200 m elevation) in August 2019. Three 15-month-old male Yunnan semi-fine wool sheep (55 ± 2.3 kg of body weight) with permanent rumen cannulas were used as experimental animals. All experimental sheep were housed in individual pens and fed twice per day (0700 and 1700 h) and had free access to water and diets. The basal diet was formulated according to the recommendation of National Research Council (2007) for growing sheep except for trace elements, and the composition and nutrient levels are shown in Table 1. The coated trace elements (contained 120 mg Fe, 16 mg Cu, 50 mg Mn, 52 mg Zn, 0.8 mg Se, 1.0 mg I and 0.8 mg Co per gram) were commercially produced and provided by Fujian Syno Biotech Co., Ltd. (Fujian, China). The final product of coated trace elements is a molten granulate produced by proportioning an inorganic trace element premix and basic coating materials (carboxymethyl cellulose, resistant starch and maltodextrin) in the solid phase. Approximately 5.0 g of coated trace elements was added to a heat-sealed nylon bag (4 × 6 cm in bag size; 50 μm in pore size). Each sheep was considered a replicate, each sample was repeated three times, and each sample had two parallel replicates per sheep for a total of six replicates. A maximum of four bags with samples were placed in the liquid phase of the rumen contents through a polyethylene hose at the same time. The coated trace elements were sequentially incubated in different batches for 0, 2, 4, 8, 16, 24, 36, and 48 h.

**TABLE 1:** Ingredients and chemical composition of basal diet (DM basis).

Ingredients	Content, %	Chemical composition^b^	
Corn	36.00	Metabolic energy, MJ/kg	9.36
Soybean meal	7.80	Crude protein, %	10.78
Wheat bran	5.10	Neutral detergent fiber, %	36.24
NaCl	0.22	Acid detergent fiber, %	18.77
NaHCO_3_	0.38	Calcium, %	0.48
Premix^a^	0.50	Phosphorus, %	0.39
Corn silage	30.00	Iron, mg/kg	84.61
Wheat straw	20.00	Manganese, mg/kg	5.23
Total	100.00	Zinc, mg/kg	25.47
		Copper, mg/kg	18.65
		Selenium, mg/kg	0.02
		Iodine, mg/kg	0.09
		Cobalt, mg/kg	0.05

^a^The premix provided the following per kg of diet: vitamin A, 10,000 IU; vitamin D_3_, 1,000 IU; vitamin E 33.4 mg.

^b^Calculated values.

**Exp. 2.** The experiment was performed at the same farm as Exp. 1 from August to September 2021. A total of thirty 4-month-old male Yunnan semi-fine wool sheep (29.7 ± 0.3 kg of body weight) were divided into three treatments in a completely randomized design as follows: CON treatment, fed with a basal diet without adding exogenous trace elements; CTE treatment, fed with a basal diet plus 400 mg/kg coated trace elements in Exp. 1; UTE treatment, fed with a basal diet plus an equal amount of trace elements with uncoated form. The basal diet was kept the same as that in Exp. 1. The addition of 400 mg/kg coated trace elements had the best growth performance of Yunnan semi-fine wool sheep, according to our previous study (unpublished data). The uncoated trace elements are constituted by ferrous sulfate heptahydrate, copper sulfate pentahydrate, manganese sulfate monohydrate, zinc sulfate heptahydrate, sodium selenite pentahydrate, potassium iodide, and cobalt chloride hexahydrate. All experimental sheep were housed in separate pens and fed twice daily at 0700 and 1700 h (allowed 5% refusal). The trial lasted for 40 days, of which 10 days were the adaptation period and 30 days were the formal trial period.

### 2.3 Sample collection

All mobile nylon bags were incubated at the same time (0630 h) prior to morning feeding and retrieved according to the expected incubation time in Exp. 1. After removal from the rumen, the nylon bags were repeatedly rinsed under cold running water to remove adhesions and subsequently placed in cold water for soaking while the nylon bags were repeatedly kneaded by hand gently until the filtered water was clear (Wang E. et al., 2021). The cleaned nylon bags were dried to constant weight at 65°C for 48 hours, and the weight after drying was recorded and the rumen disappearance rate of the coated trace elements was calculated by the difference from the initial weight.

In Exp. 2, the body weight of experimental sheep was determined before the morning feeding at the beginning (day 0) and end (day 30) of the experiment to calculate the average daily gain (ADG). The orts were collected and weighed every day, the dry matter intake (DMI) per sheep was obtained from the ration supplied and refused, and the feed conversion ratio (FCR) was calculated as dividing DMI by ADG. Four sheep were randomly selected from each group and housed in individual metabolic cages for 7 days (from day 10 to 16, the first two days were the adaptation period), and all feces were collected, weighed and recorded. A 5% aliquot of each fecal sample was removed and immediately stored at −20°C until further analysis. Jugular vein blood samples were collected on day 30 before feeding in the morning and centrifuged at 1,600 × g for 10 minutes to obtain serum, which were then aliquoted and stored at −20°C until the next analysis. On day 30, four sheep of close to group average weight in each treatment were slaughtered, and tissue samples of duodenum, jejunum and ileum and chyme samples of ileum and colon were collected. For the tissue samples, the 2- and 6-cm segments were cut at mid-duodenum, mid-jejunum and mid-ileum, respectively (Hou et al., 2010). The 2-cm intestinal segments were flushed gently with pre-cooled 0.9% sodium chloride solution (w/v, physiological saline, Sichuan Kelun Pharmaceutical Co., Ltd., Chengdu, China) and then placed in 4% paraformaldehyde fix solution (Beyotime, Shanghai, China) for morphological analysis. The 6-cm intestinal segments were opened longitudinally and the contents were flushed with pre-cooled physiological saline and immediately frozen in liquid nitrogen for total RNA extraction. Chyme from the mid-section of the ileum and colon was collected in 2 mL sterile cryopreserved tubes (Wuxi NEST Biotechnology Co., Ltd., Wuxi, China) for bacterial DNA extraction. All sampling procedure were finished within 30 minutes after the sheep being slaughtered.

### 2.4 Total tract apparent nutrient digestibility

Fecal samples from each sheep were thawed and mixed together, then dried in a forced air oven at 55°C for 48 hours and ground to pass through a 40-mesh screen before analysis, as were the samples of diet and orts. The content of dry matter (DM), crude protein (CP), acid detergent fiber (ADF), calcium (Ca) and phosphorus (P) was analyzed according to AOAC (AOAC International, 2007), and the content of neutral detergent fiber (NDF) were determined according to van Soest et al. (1991). Consequently, the apparent digestibility of nutrients can be calculated as follow: apparent nutrient digestibility = [(feed intake × nutrient content in the diet) − (fecal output × nutrient content in the feces)]/(feed intake × nutrient content in the diet × 100% (Stein and Bohlke, 2007).

### 2.5 Intestinal morphological observation

The 2-cm intestinal segments for morphological analysis were dehydrated and embedded in paraffin, cut into three sections with 5 μm thick and stained with Hematoxylin and Eosin (H&E). Morphological observation was carried out with the Nikon ECLIPSE 80i microscope (Nikon Co., Ltd., Tokyo, Japan). Villus height (distance from villus tip to crypt ostia) and width (distance from widest villus), crypt depth (distance from crypt ostia to base) were measured with the Image-Pro Plus 6.0 software (Media Cybernetics Inc., Bethesda, MD, United States). The ratio of villus height and crypt depth (V/C) was calculated as dividing villus height by crypt depth. Only the villi heights and crypt depths from vertically oriented ten adjacent villi were measured and averaged (Hou et al., 2010).

### 2.6 Serum sample analysis

The serum concentrations of Fe, Cu, Mn, Zn, Se, I and Co were determined in an induced coupled plasma mass-spectrometry (iCAP™ RQ ICP-MS, Thermo Fisher Scientific, Waltham, MA, USA) as described previously with minor modifications (Liu et al., 2014). In brief, 0.1 mL of reconstituted serum samples were diluted to 2 mL with a 1: 20 (v/v) sample diluent solution [constructed from 0.01% (w/v) Triton X-100, 0.005% (w/v) L-cysteine, 0.5% (v/v) nitric acid and deionized water]. The standard solutions containing the required seven single elements were purchased from the TMRM QC standards material center (Changzhou, China), and then diluted with sample diluent solution to appropriate concentrations according to the predicted concentrations of trace elements in serum. The major ICP-MS operating parameters were set as follows: Analyzer Pressure Readback, 1E-06 mbar; Spray Chamber Temperature, 2°C; Cool Flow, 14 L/min; Auxilliary Flow, 0.79 L/min; Nebulizer Flow, 1.06 L/min; Plasma Power, 1550 W; Q Cell gas, He 4.0 mL/min; injection method, automatic injection.

### 2.7 Real-time quantitative PCR (RT-PCR) analysis

Intestinal tissue samples were ground into powder in liquid nitrogen. Total RNA was extracted from tissue samples with the RNA extraction and purification kit (Tiangen Biotech Co. Ltd., Beijing, China) according to the manufacturer’s instructions. The concentration and purity of total RNA were determined with the NanoDrop 2000 spectrophotometer (Thermo Fisher Scientific, Wilmington, DE, USA). The integrity of total RNA was assessed by the 1% denatured agarose gel electrophoresis. Subsequently, the qualified total RNA was reverse transcribed to cDNA by a reverse transcription Kit (HiScript^®^ III RT SuperMix for qPCR (+gDNA wiper), Vazyme Biotech Co., Ltd., Nanjing, China). The real-time quantitative PCR (RT-PCR) reaction was performed with a real-time quantitative PCR Kit (ChamQ SYBR Color qPCR Master Mix (low ROX premixed), Vazyme Biotech Co., Ltd., Nanjing, China) in a QuantStudio™ 6 Real-Time PCR system (Applied Biosystems, Foster City, CA, USA) following the manufacturer’s instruction. Relative expression of genes was calculated by using the comparative cycle threshold (2^–ΔΔCt^) method. The mean value of gene expression levels in the CON treatment was set to 1.00, and GAPDH was used as internal control. The sequence and product size of genes used in this study are shown in Table 2.

**TABLE 2:** The sequence and product size of genes.

Gene	Sequence (5′–3′)	Accession ID	Product length (bp)
*Occludin*	F: CCGTTGACTTCACCTGTGGA	XM_012096797.4	195
	R: TTCCCTGATCCAGTCGTCCT		
*Claudin-1*	F: AAGACGACGAGGCACAGAAG	NM_001185016.1	186
	R: CAGCCCAGCCAATGAAGAGA		
*Claudin-2*	F: CCTCCCTGTTCTCCCTGGTA	XM_027963236.2	299
	R: GCACCTTCTGACACGATCCA		
*ZO-1*	F: GATGTTGCCAGGGAGAAGCT	XM_042235171.1	247
	R: TCACACCCTGCTTTGAGTCC		
*ZO-2*	F: TCCTTGTGAGTGGGATTGGC	XM_027964513.2	365
	R: TCTTCCCGCTTTTCCTCAGC		
*ZNT4*	F: GCCTTTGACTTCTCGGACGA	XM_042252522.1	175
	R: TGTTGGCCAAGGGTAAGTCC		
*FPN1*	F: CAGGGAGGATGCTGTGGATC	XM_042243635.1	216
	R: GTCGCCAATGATAGCTCCCA		
*GAPDH*	F: GGTCACCAGGGCTGCTTTTA	NM_001190390.1	147
	R: TTCCCGTTCTCTGCCTTGAC		

### 2.8 DNA Extraction, amplification, and sequencing

The microbial diversity in ileal and colonic chyme was detected by 16s rDNA sequencing. The total genome DNA (gDNA) from the chyle samples was extracted and purified with the ZymoBIOMICS™ DNA Microprep Kit (Zymo Research, Orange, CA, USA). The NanoDrop 2000 spectrophotometer (Thermo Fisher Scientific) and 0.8% agarose gel electrophoresis were used to detect the concentration and integrity of DNA, respectively. The universal bacterial primer (515F: 5′-GTGYCAGCMGCCGCGGTAA-3′ and 806R: 5′-GGACTACHVGGGTWTCTAAT-3′) with sequencing adapter and barcode were used to amplified the V3-V4 regions of the 16S rRNA gene (Caporaso et al., 2011). The PCR reaction was conducted in the GeneAmp PCR system 9700 thermocycler (Applied Biosystem, Foster city, CA, USA) with the high-fidelity polymerase KOD-401B FX Neo (Toyobo, Osaka, Japan) and the program was as follows: 94°C for 1 min, followed by 30 cycles at 94°C for 20 s, 54°C for 30 s and 72°C for 30 s, and a final extension at 72°C for 5 min. The resulting PCR products were extracted from a 2% agarose gel and further purified with the Zymoclean Gel Recovery Kit (Zymo Research) and quantified with the Qubit 2.0 Fluorometer (Thermo Fisher Scientific, Waltham, MA, USA). Sequencing by synthesis was performed with the HiSeq Rapid SBS kit v2 (200 cycles, Illumina, Inc., San Diego, CA, USA) on the Illumina HiSeq2500 platform at Chengdu Rhonin Biosciences Co., Ltd. (Chengdu, China).

### 2.9 Sequencing data analyses

The processing of sequencing data was performed as previously described (Zhou et al., 2022). Briefly, the Fast Length Adjustment of SHort reads (FLASH v1.2.11)^1^ were used to splice the paired-end sequencing reads obtained from the Illumina platform, which then assigned to samples based on the unique barcode 37. The raw data was generated by truncating barcode sequences, followed by trimming using Trimmomatic v0.36,^2^ and using the Uchime algorithm^3^ to identify and remove chimera sequences based on the “Gold” database^4^ to obtain clean reads (Edgar et al., 2011; Bolger et al., 2014). The qualified reads were clustered into operational taxonomic units (OTUs) by using the Usearch (a free unique sequence analysis tool)^5^ based on a 97% similarity threshold (Edgar, 2013). The analysis of α-diversity (including Shannon, Simpson, Chao1, ACE, the Good’s coverage, and PD indexes) and β-diversity as well as principal coordinate analysis (PCoA) of the Bray-Curtis distance were performed using the R package (Kembel et al., 2010; Chen et al., 2012). The potential Kyoto Encyclopedia of Genes and Genomes (KEGG) functional profiles of microbial communities were predicted with Tax4Fun^6^ (a free software package that predicts the functional capabilities of microbial communities based on 16S rRNA datasets) based on the SILVA ribosomal RNA database,^7^ and the secondary metabolic pathways were clustered (Aßhauer et al., 2015).

### 2.10 Calculations and statistical analysis

The degradation kinetic parameters of coated trace elements over time were calculated with the following exponential equation (Ørskov and McDonald, 1979): DR = a + b (1– e ^–c×t^), where “DR” is the disappearance rate of coated trace elements at time “t” (%), “a” is the rapidly degradable fraction (%), “b” is the potentially degradable fraction (%) and “c” is the rate at which b is degraded per hour (rate constant). The values of “a,” “b,” and “c” were calculated with the non-linear procedure of SPSS v23.0 (SPSS Inc., Chicago, IL, USA). The effective degradability (ED, %) was calculated according to (Ørskov and McDonald, 1979): ED = a + [b × c/(c + k)], where “a,” “b,” and “c” are the same parameters as above, “k” is the rumen particle passage rate (%/h). When fed *ad libitum*, the value of “k” was 0.06, according to (Ørskov and McDonald, 1979).

The experimental data in Exp. 2 were statistically analyzed with one-way analysis of variance (ANOVA) followed Duncan’s *post-hoc* test or Kruskal–Wallis-test with SPSS version 23.0. Each sheep was regarded as a statistical unit. *P* ≤ 0.05 was considered statistically significant, 0.05 < *P* < 0.10 was considered as trends. The results were shown as mean and standard error (SEM).

## 3 Results

### 3.1 Rumen degradation rate

**FIGURE 1:**
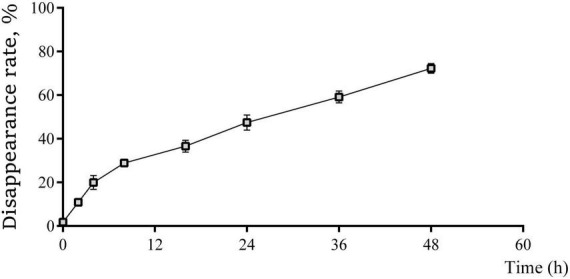
The real-time rumen disappearance rate of coated trace elements within 48 h of culture in nylon bag. Data are represented as mean and standard deviation, *n* = 3.

The ruminal disappearance rates of coated trace elements at different times are shown in Figure 1. The non-linear model resulted in the degradation kinetic parameters as follow: a = 6.292, b = 81.728, c = 0.031 and R^2^ = 0.974. Thus, the rumen effective degradability of coated trace elements can be calculated as 34.13%, the rumen passage rate was 65.87%.

### 3.2 Growth performance

Compared with the CON treatment, the final body weight and ADG were increased (*P* < 0.05) in the CTE and UTE treatments, the FCR was improved (*P* < 0.05) in the CTE and UTE treatments, whereas there was no difference between the CTE and UTE treatments (Table 3).

**TABLE 3:** Effects of coated and uncoated trace elements on growth performance of sheep.

Item	Treatments^1^			SEM	*P*-value
	CON	CTE	UTE		
Initial body weight, kg	29.76	29.78	29.79	0.45	1.000
Final body weight, kg	35.80^a^	37.81^b^	37.79^b^	0.39	0.040
Average daily gain, g	201.24^a^	267.77^b^	266.80^b^	11.51	0.020
Dry matter intake, kg	1.29	1.32	1.25	0.02	0.255
FCR	6.45^b^	5.32^a^	4.89^a^	0.25	0.022

^1^CON, sheep fed with the basal diets; CTE, sheep fed with the basal diets plus 400 mg/kg coated trace elements; UTE, sheep fed with the basal diets plus an equal amount of trace elements in uncoated form; FCR, feed conversion ratio. Data with different small letter superscripts mean significantly different (*P* < 0.05), *n* = 10.

### 3.3 Total tract apparent nutrient digestibility

The apparent digestibility of CP was higher (*P* < 0.05) in the CTE and UTE treatments than that in the CON treatment, and that of other nutrients was not affected by the addition of trace elements (Table 4).

**TABLE 4:** Effects of coated and uncoated trace elements on total tract apparent digestibility of nutrients.

Item	Treatments^1^			SEM	*P*-value
	CON	CTE	UTE		
Dry matter, %	57.28	63.10	61.53	1.13	0.078
Crude protein, %	47.87^a^	59.88^b^	60.64^b^	2.04	0.002
Neutral detergent fiber, %	42.16	52.64	51.70	2.11	0.064
Acid detergent fiber, %	50.37	49.87	50.82	1.22	0.960
Calcium, %	54.51	55.56	56.79	1.04	0.713
Phosphorus, %	44.66	50.44	46.71	1.69	0.406

^1^CON, sheep fed with the basal diets; CTE, sheep fed with the basal diets plus 400 mg/kg coated trace elements; UTE, sheep fed with the basal diets plus an equal amount of trace elements in uncoated form. Data with different small letter superscripts mean significantly different (*P* < 0.05), *n* = 4.

### 3.4 Serum trace elements

Compared with the CTE treatment, the serum levels of Se and Co were lower (*P* < 0.05) in both the CON and UTE treatments, the serum level of I was lower (*P* < 0.05) in the CON treatment (Table 5).

**TABLE 5:** Effects of coated and uncoated trace elements on serum content of trace element.

Item	Treatments^1^			SEM	*P*-value
	CON	CTE	UTE		
Iron, μg/L	3368.30	4081.05	3321.20	207.14	0.357
Copper, μg/L	668.01	658.55	646.68	26.25	0.956
Manganese, μg/L	6.71	12.01	9.04	1.02	0.092
Zinc, μg/L	709.65	842.16	780.21	24.45	0.071
Selenium, μg/L	74.04^a^	107.76^b^	65.82^a^	7.51	0.033
Iodin, μg/L	80.57^a^	99.24^b^	87.65^ab^	3.31	0.047
Cobalt, μg/L	0.41^a^	1.48^b^	0.63^a^	0.14	<0.001

^1^CON, sheep fed with the basal diets; CTE, sheep fed with the basal diets plus 400 mg/kg coated trace elements; UTE, sheep fed with the basal diets plus an equal amount of trace elements in uncoated form. Data with different small letter superscripts mean significantly different (*P* < 0.05), *n* = 4.

### 3.5 Intestinal morphology

Dietary coated trace elements increased (*P* < 0.05) the villus height in the duodenum, jejunum and ileum, increased (*P* < 0.05) the crypt depth in the jejunum and the V/C in the ileum. No difference in intestinal morphology was observed between UTE and CON treatments (Table 6).

**TABLE 6:** Effects of coated and uncoated trace elements on intestinal morphology.

Item	Treatments^1^			SEM	*P*-value
	CON	CTE	UTE		
**Duodenum**					
Villus height, mm	478.35^a^	688.66^b^	505.33^a^	11.85	<0.001
Crypt depth, mm	228.91	242.74	232.17	3.92	0.325
V/C	2.12^a^	2.90^b^	2.23^a^	0.06	<0.001
**Jejunum**					
Villus height, mm	426.04^a^	612.10^b^	443.61^a^	9.89	<0.001
Crypt depth, mm	189.28^a^	261.95^b^	200.63^a^	5.22	<0.001
V/C	2.31	2.39	2.27	0.04	0.448
**lleum**					
Villus height, mm	290.57^a^	457.81^b^	297.92^a^	9.29	<0.001
Crypt depth, mm	194.52	205.86	189.42	3.46	0.139
V/C	1.52^a^	2.29^b^	1.63^a^	0.05	<0.001

^1^CON, sheep fed with the basal diets; CTE, sheep fed with the basal diets plus 400 mg/kg coated trace elements; UTE, sheep fed with the basal diets plus an equal amount of trace elements in uncoated form; V/C, the ratio of villus height and crypt depth. Data with different small letter superscripts mean significantly different (*P* < 0.05), *n* = 4.

### 3.6 Relative gene expression of tight junction protein

**FIGURE 2:**
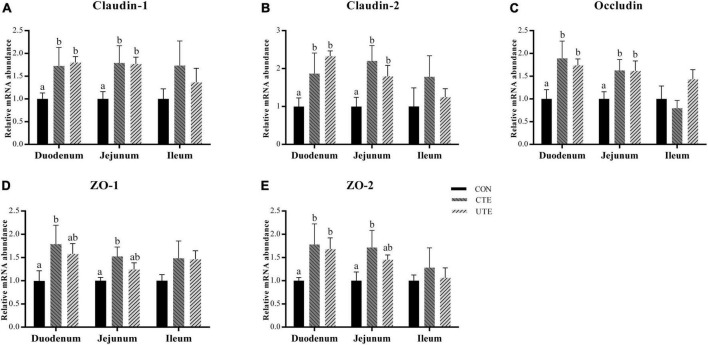
Effects of coated and uncoated trace elements on the relative gene expression of *Claudin-1* **(A)**, *Claudin-2* **(B)**, *Occludin* **(C)**, *ZO-1* **(D)**, and *ZO-2* **(E)** in small intestine. CON, sheep fed with the basal diets; CTE, sheep fed with the basal diets plus 400 mg/kg coated trace elements; UTE, sheep fed with the basal diets plus an equal amount of trace elements in uncoated form. Data are represented as mean and standard error and with different small letter superscripts mean significantly different (*P* < 0.05), *n* = 4.

As shown in Figure 2, compared with the CON treatment, both supplementation of coated and uncoated trace elements upregulated (*P* < 0.05) the relative mRNA abundance of *Claudin-1*, *Claudin-2*, and *Occludin* in the duodenum and jejunum. Dietary coated trace elements upregulated (*P* < 0.05) the mRNA abundance of *ZO-1* and *ZO-2* in the duodenum and jejunum, dietary uncoated trace elements upregulated (*P* < 0.05) the mRNA abundance of *ZO-2* in the duodenum.

### 3.7 Relative gene expression of metal ion transporters

**FIGURE 3:**
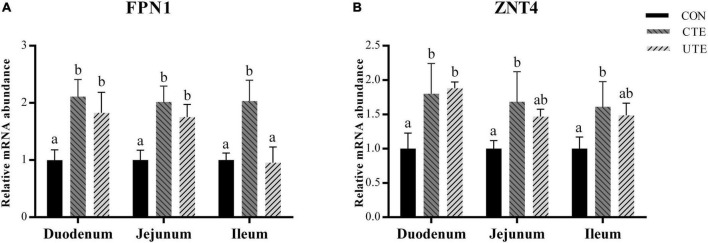
Effects of coated and uncoated trace elements on the relative gene expression of ferroportin 1 (*FPN1*, **A**) and zinc transporter 4 (*ZNT4*, **B**) in small intestine. CON, sheep fed with the basal diets; CTE, sheep fed with the basal diets plus 400 mg/kg coated trace elements; UTE, sheep fed with the basal diets plus an equal amount of trace elements in uncoated form. Data are represented as mean and standard error and with different small letter superscripts mean significantly different (*P* < 0.05), *n* = 4.

The mRNA abundance of *FPN1* and *ZNT4* in the duodenum, jejunum and ileum was higher (*P* < 0.05) in the CTE treatment than that in the CON treatment, the mRNA abundance of *FPN1* and *ZNT4* in the duodenum and *FPN1* in the jejunum was higher (*P* < 0.05) in the UTE treatment than that in the CON treatment (Figure 3).

### 3.8 Sequencing data, α-diversity, and β-diversity analysis

**FIGURE 4:**
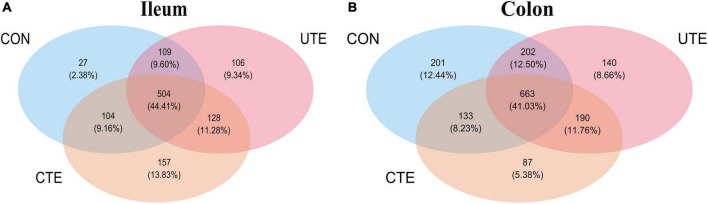
Venn diagram of the OTUs among the three treatments of bacteria in the ileum **(A)** and colon **(B)**. CON, sheep fed with the basal diets; CTE, sheep fed with the basal diets plus 400 mg/kg coated trace elements; UTE, sheep fed with the basal diets plus an equal amount of trace elements in uncoated form. *n* = 4.

**FIGURE 5:**
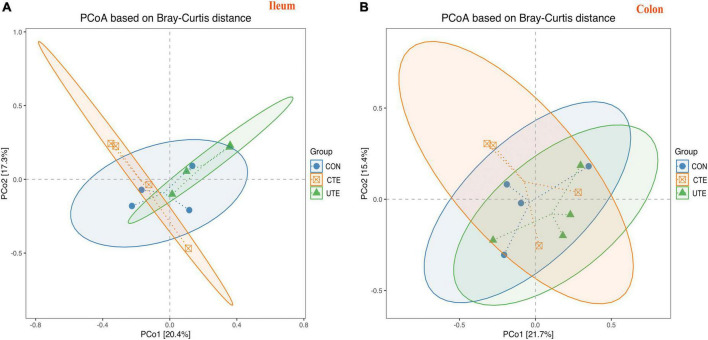
Bray–Curtis distance matrix PCoA of bacterial samples in the ileum **(A)** and colon **(B)**. CON, sheep fed with the basal diets; CTE, sheep fed with the basal diets plus 400 mg/kg coated trace elements; UTE, sheep fed with the basal diets plus an equal amount of trace elements in uncoated form. *n* = 4.

A total of 409,756 valid sequences in ileal chyme samples and 403,426 valid sequences in colon chyme samples which met quality control were obtained from the 16S rRNA high-throughput sequencing analysis. Based on 97% sequence similarity from valid sequences, these sequences were completely clustered into 9,936 OTUs in ileal chyme samples and 13,868 OTUs in colon chyme samples. The sequencing data information of bacteria in intestinal samples was shown in Supplementary Table 1. A total of 504 OTUs in ileum chyme samples were shared across the three treatments, and the number of sequences in shared OTUs accounted for 44.41% of the total effective sequences (Figure 4A). A total of 663 OTUs in colon chyme samples were shared across the three treatments, and the number of sequences in shared OTUs accounted for 44.41% of the total effective sequences (Figure 4B). The effect of coated and uncoated trace elements on α-diversity index of bacteria was shown in Supplementary Table 2. The Good’s coverage index was greater than 0.990, indicating that the sampling had sufficient sequence coverage to detect the majority of rumen bacteria. The indices of Shannon, Simpson, Chao1, ACE, PD in the ileal chyme samples were higher (*P* < 0.05) in the CTE treatment than those in the CON treatment, indicating that dietary coated trace elements increased bacterial diversity in the ileum. There was no difference in the α-diversity index of bacteria in the colon chyme among the three groups, indicating that dietary coating or uncoated micronutrients had no effect on bacterial diversity in colon chyme. The percent variation in the ileum chyme samples was represented by PCo1 (20.9%) and PCo2 (17.3%), the percent variation in the colon chyme samples was represented by PCo1 (21.7%) and PCo2 (15.4%); the closer the distance in the figure, the more similar the bacterial community composition of the samples (Figure 5).

### 3.9 Bacteria abundance at phylum and genus levels

**FIGURE 6:**
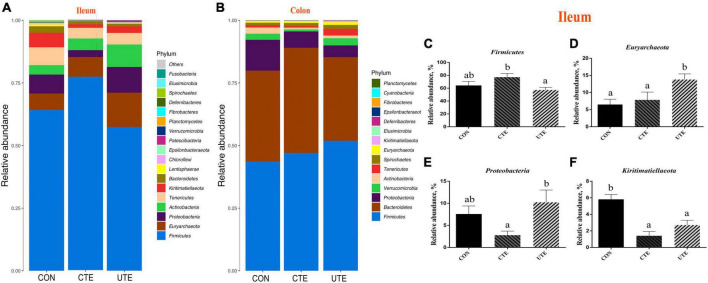
Effects of coated and uncoated trace elements on the relative abundance of bacteria at phylum level. **(A)** The bacterial composition at phylum level in the ileum; **(B)** the bacterial composition at phylum level in the colon; **(C–F)** bacteria with differences in abundance in the ileum at the phylum level. CON, sheep fed with the basal diets; CTE, sheep fed with the basal diets plus 400 mg/kg coated trace elements; UTE, sheep fed with the basal diets plus an equal amount of trace elements in uncoated form. Data are represented as mean and standard error and with different small letter superscripts mean significantly different (*P* < 0.05), *n* = 4.

There were 20 and 16 phyla taxonomically classified in the ileum and colon chyme samples, respectively. The dominant phyla of ileum bacteria were *Firmicutes* (57.28–77.24%) and *Euryarchaeota* (6.50–13.81%) (Figure 6A), and the dominant phyla of colon bacteria were *Firmicutes* (43.64–51.87%) and *Bacteroidetes* (33.39–42.06%) (Figure 6B). The relative abundance of phylum *Firmicutes* in the ileum was higher (*P* < 0.05) in the CTE treatment than that in the UTE treatment (Figure 6C). The relative abundance of phylum *Euryarchaeota* in the ileum was higher (*P* < 0.05) in the UTE treatment than that in the CON and CTE treatments (Figure 6D). The relative abundance of phylum *Proteobacteria* in the ileum was lower (*P* < 0.05) in the CTE treatment than that in the UTE treatment (Figure 6E). The relative abundance of phylum *Kiritimatiellaeota* in the ileum was higher (*P* < 0.05) in the CON treatment than that in the CTE and UTE treatments (Figure 6F).

**FIGURE 7:**
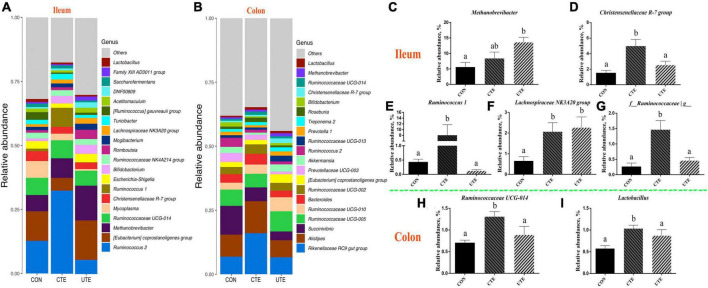
Effects of coated and uncoated trace elements on the relative abundance of bacteria at genus level. **(A)** The bacterial composition at genus level in the ileum; **(B)** the bacterial composition at genus level in the colon; **(C–G)** bacteria with differences in abundance in the ileum at the phylum level; **(H–I)** bacteria with differences in abundance in the colon at the phylum level. CON, sheep fed with the basal diets; CTE, sheep fed with the basal diets plus 400 mg/kg coated trace elements; UTE, sheep fed with the basal diets plus an equal amount of trace elements in uncoated form. Data are represented as mean and standard error and with different small letter superscripts mean significantly different (*P* < 0.05), *n* = 4.

There were 239 and 188 genera taxonomically classified in the ileum and colon chyme samples, respectively. The top three dominant genera of ileum bacteria were *Ruminococcus 2* (5.53–37.14%), *Eubacterium* (6.22–15.42%) and *Methanobrevibacter* (5.60–13.59%), as shown in Figure 7A, and the top three dominant genera of colon bacteria were *Rikenellaceae RC9 gut group* (6.72–15.97%), *Alistipes* (6.64–12.56%) and *Succinivibrio* (3.41–11.25%), as shown in Figure 7B. The relative abundance of genus *Methanobrevibacter* in the ileum was higher (*P* < 0.05) in the UTE treatment than that in the CON treatment (Figure 7C). The relative abundance of genera *Christensenellaceae R-7group*, *Ruminococcus 1* and *f__Ruminococcaceae/g*_ in the ileum was higher (*P* < 0.05) in the CTE treatment than that in the CON and UTE treatments (Figures 6D, E, G). The relative abundance of genus *Lachnospiraceae NK3A20 group* in the ileum was higher (*P* < 0.05) in the CON treatment than that in the CTE and UTE treatments (Figure 6F). The relative abundance of genus *Ruminococcaceae UCG-014* and *Lactobacillus* in the colon was higher (*P* < 0.05) in the CTE treatment than that in the CON and UTE treatments (Figures 6H, I).

### 3.10 Prediction of bacterial function

**FIGURE 8:**
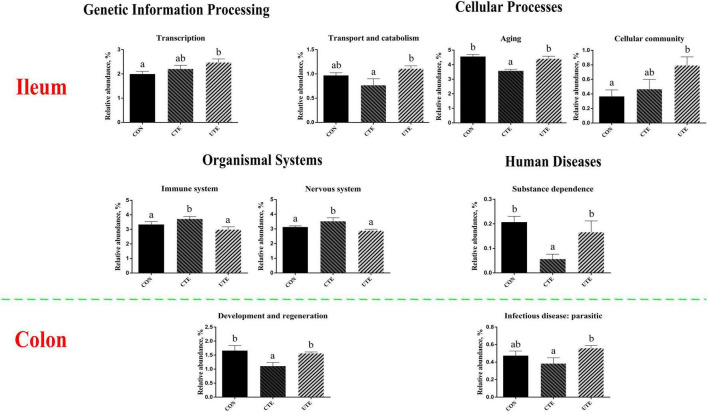
The prediction of bacterial function based on Kyoto Encyclopedia of Genes and Genomes (KEGG) pathway at level 2. CON, sheep fed with the basal diets; CTE, sheep fed with the basal diets plus 400 mg/kg coated trace elements; UTE, sheep fed with the basal diets plus an equal amount of trace elements in uncoated form. Data are represented as mean and standard error and with different small letter superscripts mean significantly different (*P* < 0.05), *n* = 4.

The effect of coated and uncoated trace elements on the prediction of function of bacteria in the ileum and colon were shown in Supplementary Tables 3, 4. The main gene functions of bacteria in the ileum associated with translation (∼5.7%), replication and repair (∼5.5%) and drug resistance: antineoplastic (∼5.0%), as shown in Supplementary Table 3. The main gene functions of bacteria in the colon are associated with immune system (∼5.2%), replication and repair (∼5.0%) and drug resistance: antineoplastic (∼5.0%), as shown in Supplementary Table 4. As shown in Figure 8, the relative abundance of transcription and cellular community of bacteria in the ileum was higher (*P* < 0.05) in the UTE treatment than that in the CON treatment, the relative abundance of transport and catabolism of bacteria in the ileum was higher (*P* < 0.05) in the UTE treatment than that in the CTE treatment. Compared with the CON and UTE treatments, the relative abundance of aging and substance dependence of bacteria in the ileum was lower (*P* < 0.05) in the UTE treatment, and the relative abundance of immune system and nervous system of bacteria in the ileum was higher (*P* < 0.05) in the UTE treatment. For the gene functions of bacteria in the colon, the relative abundance of development and regeneration was lower (*P* < 0.05) in the CTE treatment than that in the CON and UTE treatments, the relative abundance of infectious disease: parasitic was lower (*P* < 0.05) in the CTE treatment than that in the UTE treatment.

## 4 Discussion

Due to its easier absorption, it is generally accepted that organic trace elements are used more efficiently than inorganic trace elements for ruminants (Byrne and Murphy, 2022). Besides organic trace elements, coating technique may be an effective measure to improve the bioavailability of inorganic trace elements. In the current study, the effective degradability of the coated trace elements was determined to be 34.13% in Exp. 1, indicating that most of them (65.87%) could escape from the rumen and enter the subsequent gastrointestinal tract. In Exp. 2, the ADG of sheep fed the basal diet without the addition of exogenous trace elements was significantly lower than that of sheep fed the basal diet with coated or uncoated trace elements. This result may be caused by insufficient trace elements in the basal diet (mainly composed of corn, soybean meal, heat bran, corn silage and wheat straw) to meet the requirements of the growing Yunnan semi-fine wool sheep. There is well-established evidence that the deficiency of trace elements can negatively affect the growth of animals (Prasad, 1995; Eder et al., 1996; Kegley et al., 2016; Huang et al., 2017), as trace elements are involved in maintaining normal intestinal absorption and microbial homeostasis (Pajarillo et al., 2021; Skalny et al., 2021). The measurement of serum mineral concentrations is a popular and potentially valuable method for evaluating the trace mineral nutritional status and the mineral bioavailability of animals (Herdt and Hoff, 2011; Burrow et al., 2020). Thus, we measured the levels of trace elements in serum and found that the levels of Se, Co and I in the serum of sheep supplemented with coated trace elements were increased compared to CON and UTE treatments. The rumen binding of abundant fiber components and minerals in ruminant feeds may affect the subsequent absorption of some trace elements in the gut (Whitehead et al., 1985). Under the present experimental conditions, increasing the rumen passage rate appears to be an effective strategy to improve the bioavailability of trace elements. Compared with the addition of coated trace elements, the growth performance of sheep fed with uncoated trace elements did not show an advantage. This may be due to the fact that trace elements added as salt also provide sufficient amounts of minerals to growing sheep, after all, they remain the main mineral providers in livestock feed, albeit with relatively low bioavailability.

Some researchers believe that in ruminants, trace elements in the form of salts are more soluble in the rumen, chemical forms of trace mineral elements can be altered, and adverse reactions between rumen contents can affect rumen fermentation patterns, potentially affecting digestibility of nutrients (Galbraith et al., 2016; Arce-Cordero et al., 2020; Miller et al., 2020). In recent years, attempts have been made to replace mineral salts with trace elements in their hydroxyl or coating form. Addition of hydroxy trace minerals improved the digestibility of nutrients in dairy cows (Miller et al., 2020; Guimaraes et al., 2022) and steers (Caldera et al., 2019; Guimaraes et al., 2021) compared to supplementation of sulfate-sourced trace elements. However, Miller et al. (2020) reported that the source of trace mineral (sulfate or hydroxy source) had no effect on rumen turnover, and particle passage rates in Holstein cows. Arce-Cordero et al. (2020) also reported that dietary rumen-protected copper sulfate did not affect the ruminal digestibility of nutrients compared to unprotected copper sulfate. In this study, we found that the addition of coated or uncoated trace elements had no effect on apparent nutrient digestibility, with the exception of CP digestibility. The apparent digestibility of CP was higher in the CTE and UTE treatments than that in the CON treatment, which was consistent with the result of growth performance. In accordance with our findings, previous studies have shown higher digestibility of CP in dairy cows (El Ashry et al., 2012), goats (Salama et al., 2003) and growing lambs (Samarin et al., 2022) supplemented with a mix of trace minerals from different sources. Trace elements have been proven to play a critical role in regulating intestinal health and the microbiome (Shannon and Hill, 2019; Barra et al., 2021), which may be factors affecting nutrient digestibility.

The morphology of the intestinal mucosa is an important indicator of intestinal absorption function in ruminant livestock. Most absorption of trace minerals occurs in the small intestine, mainly in the duodenum, although absorption can occur anywhere in the gastrointestinal tract (Smith, 2004; Goff, 2018). The sheep in the CTE treatment had a greater villus height in the duodenum, jejunum and ileum than those sheep in the CON and UTE treatments, indicating that dietary coated trace elements were beneficial to improve the absorption function of small intestinal. Previous studies reported that a single supplement of dietary trace minerals had a positive effect on the gut health of animals, such as Fe (Zhou et al., 2020), Cu (Yin et al., 2021), Zn (Wang et al., 2018) and Se (Samo et al., 2020). Replacing inorganic trace elements with complex organic trace minerals in the diet could increase the villus height in the ileum of weaned piglets (Wang et al., 2022). The addition of coated organic trace elements could also significantly improve the villus height and villus/crypt ratio in late-phase laying hens (Chen et al., 2022). In addition to intestinal morphology, we also detected the gene expression of tight junction protein in small intestine and found that dietary coated trace elements upregulated the relative mRNA abundance of tight junction proteins (Claudin-1, Claudin-2, Occludin, ZO-1, and ZO-2) in duodenum and jejunum compared to dietary uncoated trace elements. Altering the supply form and amount of Zn could upregulate the gene expression of *Occludin* and *ZO-1* in ileal mucosa, but did not affect intestinal mucosal morphology (Wang et al., 2018). The deficiency or excess of trace elements can regulate the microenvironment of animals’ intestines, including the microbiome, nutrient availability, stress and mucosal immunity (Pajarillo et al., 2021). This could be the reason that the addition of trace elements improved the apparent digestibility of the crude protein. In this study, the supply of equal amounts of uncoated trace elements limited the biological effects in the small intestine due to low bioavailability. The absorption of metal ions by intestinal epithelial cells depends on divalent metal transporters, which are then transported to the blood by transmembrane proteins such as ferroportin and zinc transporters (Hundal and Taylor, 2009; Espinoza et al., 2012). The addition of trace elements had a positive effect on the relative mRNA abundance of iron transporter and zinc transporter (FPN1 and ZNT4), especially coated trace elements. This may be related to the amount of trace elements reaching the small intestine.

Some trace minerals ingested by animals are utilized by gut microbiota to maintain basic physiological activity, although hosts have evolved mechanisms that prevent bacteria from taking advantage of them through chelation (Becker and Skaar, 2014). Meanwhile, trace minerals also regulate the composition and diversity of intestinal microorganisms. Previous studies have reported that Fe could regulate the ability of microorganisms to obtain energy from nutrients ingested by hosts (Dostal et al., 2015), Mn could regulate the detoxification involved in reactive oxygen species, carbohydrates, lipids, and protein metabolism, and DNA replication (Kehres and Maguire, 2003; Zaharik and Brett Finlay, 2004), Zn could regulate host defenses, antioxidant systems, gene expression, virulence factors (Velasco et al., 2018; Eijkelkamp et al., 2019), and Cu could reduce the relative abundance of potential pathogens (Villagómez-Estrada et al., 2020). These are all involved in the survival and adaptation of the microbiome. In this study, the most dominant bacterial phylum in ileum and colon was *Firmicutes*, which is consistent with previous studies in sheep (Wang et al., 2017; Ma et al., 2022). Compared with the CON treatment, the addition of uncoated trace elements increased the proportion of the phylum *Euryarchaeota*, and the change of the abundance of *Kiritimatiellaeota* was the most obvious with the addition of trace elements. *Euryarchaeota* is the phylum of Archaea, including some methane-producing bacteria commonly found in the gut, known for metabolizing nutrients and metabolites of other bacteria, leading to elevated levels of short-chain fatty acids, such as acetate (Horz and Conrads, 2010; Primec et al., 2019). Dietary zinc oxide supplementation significantly increased the abundance of *Euryarchaeota* in the colon, while other information about the association between *Euryarchaeota* and trace minerals has been rarely reported. *Kiritimatiellaeota* mediated pyruvate to generate acetyl-CoA and then enter the tricarboxylic acid (TCA) cycle and involve arginine biosynthesis, and also regulated fatty acid synthesis. *Kiritimatiellaeota* mediates the breakdown of pyruvate into acetyl-CoA, which then enters the tricarboxylic acid (TCA) cycle and involves arginine biosynthesis to utilize dietary N and produce energy (Guo et al., 2021). However, the cause for the addition of trace elements to reduce the proportion of *Kiritimatiellaeota* in the ileum need to be further studied. Compared to uncoated trace elements, the addition of coated trace elements increased the proportion of the phylum *Firmicutes* while decreased the proportion of the phylum *Proteobacteria*. The proportion of dominant bacterial phyla in the ileum was significantly affected by whether or not it is coated, probably caused by the coating technique changing the amount of trace elements reaching the ileum. At the genus level, the proportion of *Christensenellaceae R-7 group*, *f_ Ruminococcaceae/g_* and *Ruminococcus 1* in the ileum was positively affected by the addition of coated trace elements. *Christensenellaceae R-7 group* play a pivotal role in the degradation of carbohydrates to acetate (Morotomi et al., 2012), and the increase in their abundance was positively correlated with the improvement of growth performance and meat quality in Hu lambs (Xiong et al., 2021). Both *f_ Ruminococcaceae/g_* and *Ruminococcus 1* belong to family *Ruminococcaceae*, which have great ability in the complex carbohydrates digestion to generate short chain fatty acids (SCFAs) (Xie et al., 2022). It has been reported that SCFAs can improve intestinal health by maintaining the integrity of intestinal barrier, secreting mucus and preventing inflammation (Peng et al., 2009; Lewis et al., 2010; Primec et al., 2017). The similar result appeared in the colon that the addition of coated trace elements increased the proportion of *Ruminococcaceae UCG-014*.

To further explore the role of microbiota in gut, the prediction of bacterial function based on KEGG pathway was performed. Findings of the present study revealed significant differences in bacterial function between sheep supplemented with coated trace elements or not. In the ileal microbiota of sheep fed with coated trace elements, there were more genes associated with the organism systems (immune system and nervous system), which critically influence the homeostasis of normal physiological functions of the host (Xing et al., 2019). Homeostasis of the gut microbiota play an important role in host health and aging (Han et al., 2017). The genes associated with aging were fewer in the CTE treatment than that in the UTE treatment, indicating that feeding coated trace elements is more beneficial for gut health in sheep. In the colon, the genes related to development and regeneration, infectious disease: parasitic were also fewer in the CTE treatment than that in the UTE treatment. Both substance dependence and infectious disease: parasitic belong to the biological pathway of human disease and have obvious associations with host health (Li and Agarwal, 2009). The decreased occurrence of these genes mirrors the decrease in sequences affiliated with intestinal injury, supporting the importance of these bacteria within the gut health. The limitation of this study is that metagenomic shotgun sequencing of gut digesta was not performed, which will provide more accurate and direct evidence for the function of gut microbiota in future studies.

## 5 Conclusion

In summary, most of the coated trace elements (65.87%) used in this experiment were able to escaped from the rumen. Under the dietary conditions of the current study, the addition of trace elements improved the growth performance and the apparent digestibility of CP of growing sheep. The addition of dietary coated trace elements had a more positive effect on serum trace element levels and intestinal development, although there was no difference in growth performance, compared to uncoated trace elements. Generally, this study partly revealed the advantages of coated trace elements over traditional trace elements for intestinal development in growing sheep, providing a theoretical basis for their further utilization on ruminants.

## Data availability statement

The datasets presented in this study can be found in online repositories. The names of the repository/repositories and accession number(s) can be found at: https://www.ncbi.nlm.nih.gov/, PRJNA894949.

## Ethics statement

This animal study was reviewed and approved by the Experimental Animal Committee of Animal Nutrition Institute, Sichuan Agricultural University.

## Author contributions

JZ and YR wrote the original draft, performed data statistical analysis, and produced figures and tables. YR and XW performed the experiments. SY, RH, QP, and YJ provided great help in data analysis. QH, JZ, and BX performed the trial investigation and design. ZW, BX, LW, HZ, and YJ reviewed and edited the draft. All authors contributed to the article and approved the submitted version.

## Footnotes

1
http://www.geneious.com/plugins/flash/

2
http://www.usadellab.org/cms/?page=trimmomatic

3
http://drive5.com/usearch/manual/uchime_algo.html

4
http://drive5.com/uchime/uchime_download.html

5
http://drive5.com/uparse/

6
http://tax4fun.gobics.de/

7
http://www.arb-silva.de/
