# Supplementary material for: Comparison of coated and uncoated trace elements on growth performance, apparent digestibility, intestinal development and microbial diversity in growing sheep
Source: Front Microbiol. 2022 Dec 20;13:1080182. doi: 10.3389/fmicb.2022.1080182 (PMC9808050; doi:10.3389/fmicb.2022.1080182)
Supplement: Supplementary file 1 [file Table_1.DOCX]

Supplementary Material

Supplementary Table S1. Effects of coated and uncoated trace elements on intestinal bacteria α-diversity index

| **Item** | **Treatments^1^** | | |
| --- | --- | --- | --- |
|  | **CON** | **CTE** | **UTE** |
| Ileum |  |  |  |
| Raw reads | 140726 | 136763 | 144127 |
| Effective reads | 135031 | 133736 | 140992 |
| Effective rate（%） | 95.95 | 97.79 | 97.82 |
| OTU counts | 2976 | 3572 | 3388 |
| Colon |  |  |  |
| Raw reads | 137219 | 134795 | 139599 |
| Effective reads | 134532 | 132220 | 136674 |
| Effective rate（%） | 98.04 | 98.09 | 97.9 |
| OTU counts | 4796 | 4292 | 4780 |

^1^CON, sheep fed with the basal diets; CTE, sheep fed with the basal diets plus 400 mg/kg coated trace elements; UTE, sheep fed with the basal diets plus an equal amount of trace elements in uncoated form.

Supplementary Table S2. Effects of coated and uncoated trace elements on intestinal bacteria α-diversity index

| **Item** | **Treatments^1^** | | | **SEM** | ***P*-Value** |
| --- | --- | --- | --- | --- | --- |
|  | **CON** | **CTE** | **UTE** |  |  |
| Ileum |  |  |  |  |  |
| Coverage | 0.996 | 0.996 | 0.996 | 0.0002 | 0.972 |
| Shannon | 3.54^b^ | 4.80^a^ | 4.13^ab^ | 0.20 | 0.019 |
| Simpson | 0.87^b^ | 0.97^a^ | 0.94^ab^ | 0.02 | 0.037 |
| Chao 1 | 422.61^b^ | 555.31^a^ | 447.40^b^ | 22.69 | 0.019 |
| ACE | 423.73^b^ | 556.25^a^ | 446.61^b^ | 22.25 | 0.014 |
| PD | 21.23^b^ | 26.65^a^ | 21.87^b^ | 0.91 | 0.010 |
| Colon |  |  |  |  |  |
| Coverage | 0.995 | 0.995 | 0.995 | 0.0003 | 0.832 |
| Shannon | 4.57 | 4.83 | 4.88 | 0.13 | 0.554 |
| Simpson | 0.95 | 0.96 | 0.98 | 0.01 | 0.537 |
| Chao 1 | 548.01 | 597.87 | 571.81 | 21.07 | 0.671 |
| ACE | 546.15 | 602.62 | 570.93 | 20.84 | 0.587 |
| PD | 25.57 | 26.92 | 27.32 | 0.53 | 0.407 |

^1^CON, sheep fed with the basal diets; CTE, sheep fed with the basal diets plus 400 mg/kg coated trace elements; UTE, sheep fed with the basal diets plus an equal amount of trace elements in uncoated form. Chao 1, the Chao1 estimator; Ace, the ACE estimator; PD, PD_ whole_ tree. Data with different small letter superscripts mean significantly different (*P* < 0.05), n = 4.

Supplementary Table S3. Effects of coated and uncoated trace elements on the prediction of bacterial function in ileum

| **Item** | **Treatments^1^** | | | **SEM** | ***P*-Value** |
| --- | --- | --- | --- | --- | --- |
|  | **CON** | **CTE** | **UTE** |  |  |
| Metabolism |  |  |  |  |  |
| Carbohydrate metabolism | 2.28 | 2.24 | 2.27 | 0.03 | 0.879 |
| Energy metabolism | 1.98 | 1.91 | 2.12 | 0.05 | 0.340 |
| Lipid metabolism | 2.09 | 2.00 | 2.09 | 0.03 | 0.449 |
| Nucleotide metabolism | 3.48 | 3.50 | 3.56 | 0.04 | 0.769 |
| Amino acid metabolism | 2.52 | 2.56 | 2.58 | 0.01 | 0.304 |
| Metabolism of other amino acids | 2.05 | 1.99 | 2.05 | 0.03 | 0.702 |
| Glycan biosynthesis and metabolism | 1.89 | 1.84 | 1.82 | 0.02 | 0.418 |
| Metabolism of cofactors and vitamins | 2.65 | 2.48 | 2.78 | 0.07 | 0.234 |
| Metabolism of terpenoids and polyketides | 1.52 | 1.53 | 1.55 | 0.02 | 0.854 |
| Biosynthesis of other secondary metabolites | 3.02 | 2.94 | 2.96 | 0.03 | 0.52 |
| Xenobiotics biodegradation and metabolism | 0.59 | 0.54 | 0.61 | 0.02 | 0.557 |
| Not included in regular maps | 2.6 | 2.99 | 2.41 | 0.12 | 0.154 |
| Genetic Information Processing |  |  |  |  |  |
| Transcription | 1.99^a^ | 2.2^ab^ | 2.47^b^ | 0.09 | 0.042 |
| Translation | 5.53 | 5.86 | 5.78 | 0.07 | 0.134 |
| Folding, sorting and degradation | 4.84 | 4.83 | 4.86 | 0.04 | 0.978 |
| Replication and repair | 5.47 | 5.64 | 5.45 | 0.05 | 0.220 |
| Environmental Information Processing |  |  |  |  |  |
| Membrane transport | 1.38 | 1.3 | 1.34 | 0.02 | 0.334 |
| Signal transduction | 1.11 | 1.06 | 1.1 | 0.03 | 0.736 |
| Signaling molecules and interaction | 0 | 0.01 | 0 | 0 | 0.317 |
| Cellular Processes |  |  |  |  |  |
| Transport and catabolism | 0.97^ab^ | 0.77^a^ | 1.11^b^ | 0.06 | 0.042 |
| Cell motility | 3.66 | 3.66 | 3.51 | 0.27 | 0.972 |
| Cell growth and death | 3.39 | 3.33 | 3.31 | 0.02 | 0.168 |
| Cellular community - eukaryotes | 0.37^a^ | 0.46^ab^ | 0.79^b^ | 0.08 | 0.048 |
| Cellular community - prokaryotes | 1.79 | 1.67 | 1.76 | 0.03 | 0.191 |
| Aging | 4.56^b^ | 3.57^a^ | 4.4^b^ | 0.15 | 0.002 |
| Organismal Systems |  |  |  |  |  |
| Immune system | 3.34^a^ | 3.71^b^ | 2.98^a^ | 0.13 | 0.049 |
| Endocrine system | 2.85 | 2.97 | 2.78 | 0.06 | 0.442 |
| Circulatory system | 0.18 | 0.03 | 0.14 | 0.03 | 0.141 |
| Digestive system | 0.98 | 1.55 | 1.02 | 0.3 | 0.726 |
| Excretory system | 1.25 | 1.21 | 0.86 | 0.18 | 0.676 |
| Nervous system | 3.13^a^ | 3.52^b^ | 2.88^a^ | 0.11 | 0.048 |
| Development and regeneration | 1.69 | 1.93 | 1.49 | 0.2 | 0.703 |
| Environmental adaptation | 2.38 | 2.27 | 2.3 | 0.05 | 0.640 |
| Human Diseases |  |  |  |  |  |
| Cancer: overview | 2.64 | 2.76 | 2.63 | 0.04 | 0.336 |
| Cancer: specific types | 1.39 | 1.1 | 1.26 | 0.07 | 0.258 |
| Immune disease | 1.94 | 1.73 | 2.44 | 0.19 | 0.315 |
| Neurodegenerative disease | 1.26 | 1.11 | 1.32 | 0.05 | 0.231 |
| Substance dependence | 0.21^b^ | 0.06^a^ | 0.17^b^ | 0.03 | 0.023 |
| Cardiovascular disease | 2.35 | 2.38 | 2.27 | 0.06 | 0.72 |
| Endocrine and metabolic disease | 3.14 | 3.31 | 3.04 | 0.07 | 0.259 |
| Infectious disease: bacterial | 1.4 | 1.39 | 1.32 | 0.02 | 0.304 |
| Infectious disease: viral | 0.84 | 0.74 | 1.03 | 0.06 | 0.172 |
| Infectious disease: parasitic | 0.9 | 1.13 | 0.9 | 0.07 | 0.314 |
| Drug resistance: antimicrobial | 1.46 | 1.37 | 1.35 | 0.03 | 0.225 |
| Drug resistance: antineoplastic | 4.95 | 4.82 | 5.15 | 0.1 | 0.422 |

^1^CON, sheep fed with the basal diets; CTE, sheep fed with the basal diets plus 400 mg/kg coated trace elements; UTE, sheep fed with the basal diets plus an equal amount of trace elements in uncoated form. Data with different small letter superscripts mean significantly different (*P* < 0.05), n = 4.

Supplementary Table S4. Effects of coated and uncoated trace elements on the prediction of bacterial function in colon

| **Item** | **Treatments^1^** | | | **SEM** | ***P*-Value** |
| --- | --- | --- | --- | --- | --- |
|  | **CON** | **CTE** | **UTE** |  |  |
| Metabolism |  |  |  |  |  |
| Carbohydrate metabolism | 2.11 | 2.16 | 2.13 | 0.02 | 0.525 |
| Energy metabolism | 1.67 | 1.70 | 1.71 | 0.02 | 0.599 |
| Lipid metabolism | 2.07 | 2.11 | 2.05 | 0.02 | 0.525 |
| Nucleotide metabolism | 3.26 | 3.26 | 3.31 | 0.02 | 0.554 |
| Amino acid metabolism | 2.42 | 2.47 | 2.44 | 0.01 | 0.146 |
| Metabolism of other amino acids | 1.93 | 1.99 | 1.94 | 0.01 | 0.214 |
| Glycan biosynthesis and metabolism | 2.85 | 2.88 | 2.67 | 0.06 | 0.392 |
| Metabolism of cofactors and vitamins | 2.50 | 2.55 | 2.51 | 0.02 | 0.520 |
| Metabolism of terpenoids and polyketides | 2.04 | 2.07 | 2.10 | 0.01 | 0.140 |
| Biosynthesis of other secondary metabolites | 3.87 | 3.95 | 3.85 | 0.03 | 0.367 |
| Xenobiotics biodegradation and metabolism | 0.55 | 0.58 | 0.58 | 0.01 | 0.785 |
| Not included in regular maps | 2.34 | 3.09 | 2.43 | 0.22 | 0.358 |
| Genetic Information Processing |  |  |  |  |  |
| Transcription | 1.35 | 1.27 | 1.37 | 0.03 | 0.381 |
| Translation | 4.68 | 4.56 | 4.77 | 0.05 | 0.194 |
| Folding, sorting and degradation | 4.18 | 4.17 | 4.26 | 0.03 | 0.423 |
| Replication and repair | 4.98 | 4.95 | 5.07 | 0.03 | 0.270 |
| Environmental Information Processing |  |  |  |  |  |
| Membrane transport | 1.09 | 1.12 | 1.12 | 0.04 | 0.949 |
| Signal transduction | 1.12 | 1.12 | 1.15 | 0.01 | 0.739 |
| Signaling molecules and interaction | 0.00 | 0.01 | 0.00 | 0.00 | 0.186 |
| Cellular Processes |  |  |  |  |  |
| Transport and catabolism | 1.78 | 1.96 | 1.68 | 0.09 | 0.443 |
| Cell motility | 3.04 | 2.64 | 2.97 | 0.15 | 0.558 |
| Cell growth and death | 3.48 | 3.49 | 3.46 | 0.02 | 0.795 |
| Cellular community - eukaryotes | 0.03 | 0.04 | 0.06 | 0.01 | 0.585 |
| Cellular community - prokaryotes | 1.80 | 1.73 | 1.80 | 0.04 | 0.770 |
| Aging | 3.98 | 4.04 | 3.90 | 0.06 | 0.695 |
| Organismal Systems |  |  |  |  |  |
| Immune system | 5.20 | 5.05 | 5.20 | 0.04 | 0.316 |
| Endocrine system | 3.39 | 3.40 | 3.44 | 0.02 | 0.733 |
| Circulatory system | 0.03 | 0.03 | 0.03 | 0.00 | 0.305 |
| Digestive system | 1.77 | 1.57 | 1.26 | 0.13 | 0.301 |
| Excretory system | 0.88 | 0.83 | 1.07 | 0.09 | 0.594 |
| Nervous system | 2.62 | 2.62 | 2.57 | 0.05 | 0.906 |
| Development and regeneration | 1.66^b^ | 1.11^a^ | 1.56^b^ | 0.10 | 0.034 |
| Environmental adaptation | 2.67 | 2.66 | 2.62 | 0.05 | 0.904 |
| Human Diseases |  |  |  |  |  |
| Cancer: overview | 2.68 | 2.73 | 2.79 | 0.04 | 0.546 |
| Cancer: specific types | 1.30 | 1.40 | 1.37 | 0.03 | 0.469 |
| Immune disease | 3.59 | 3.96 | 3.67 | 0.14 | 0.552 |
| Neurodegenerative disease | 0.78 | 0.75 | 0.70 | 0.03 | 0.605 |
| Substance dependence | 0.01 | 0.01 | 0.02 | 0.00 | 0.358 |
| Cardiovascular disease | 2.49 | 2.55 | 2.55 | 0.04 | 0.773 |
| Endocrine and metabolic disease | 2.43 | 2.33 | 2.44 | 0.03 | 0.377 |
| Infectious disease: bacterial | 1.47 | 1.41 | 1.44 | 0.02 | 0.287 |
| Infectious disease: viral | 0.57 | 0.57 | 0.58 | 0.02 | 0.953 |
| Infectious disease: parasitic | 0.47^ab^ | 0.38^a^ | 0.56^b^ | 0.03 | 0.048 |
| Drug resistance: antimicrobial | 1.81 | 1.81 | 1.78 | 0.03 | 0.899 |
| Drug resistance: antineoplastic | 5.05 | 4.94 | 5.04 | 0.05 | 0.675 |

^1^CON, sheep fed with the basal diets; CTE, sheep fed with the basal diets plus 400 mg/kg coated trace elements; UTE, sheep fed with the basal diets plus an equal amount of trace elements in uncoated form. Data with different small letter superscripts mean significantly different (*P* < 0.05), n = 4.
